# Concerns regarding tablet splitting: a systematic review

**DOI:** 10.3399/BJGPO.2022.0001

**Published:** 2022-06-01

**Authors:** Aanchal K Saran, Natalie A Holden, Scott R Garrison

**Affiliations:** 1 Department of Family Medicine, University of Alberta, Edmonton, Alberta, Canada; 2 Faculty of Pharmacy and Pharmaceutical Sciences, University of Alberta, Edmonton, Alberta, Canada

**Keywords:** cost savings, delayed-action preparations, dose-reduction, geriatric prescribing, medication errors, pill-splitting, tablets

## Abstract

**Background:**

Tablet splitting can provide dose flexibility and cost savings; however, pharmaceutical representatives typically discourage the practice.

**Aim:**

To identify and summarise all published concerns related to tablet splitting and to present the experimental evidence that investigates those concerns.

**Design & setting:**

Systematic review and qualitative synthesis of tablet-splitting concerns and evidence.

**Method:**

Medline and EMBASE databases were searched over all years of publication for articles in English discussing the splitting of tablets. Eligible articles included original research, narrative reviews, systematic reviews, and expert opinion.

**Results:**

After removing duplicates, 1837 potentially relevant articles underwent dual review, whereupon 1612 articles were excluded based on title and abstract. After examination of 225 full texts, 138 articles were included (one systematic review, four narrative reviews, 101 original research articles, and 32 opinion articles). The described concerns included difficulty breaking tablets, loss of mass, weight variability, chemical instability, overly rapid dosing if sustained-release medications are split, non-compliance, and patient confusion resulting in medication errors. No substantive evidence was found to support concerns regarding loss of mass, weight variability, chemical instability, or non-compliance. Evidence does support some older adults struggling to split tablets without tablet splitters, and the inappropriateness of splitting sustained-release preparations, given the potential for alteration of the rate of drug release for some products.

**Conclusion:**

With the exception of sustained-release tablets, which should not be split, and excepting those older people who may struggle to split tablets based on physical limitations, there is little evidence to support tablet-splitting concerns.

## How this fits in

In the authors’ experience, pharmaceutical representatives commonly discourage the splitting of their products. In the literature, concerns have been raised about difficulty breaking tablets, losing tablet mass, unequal splitting, chemical instability, confusion leading to medication errors, and the mistaken splitting of sustained-release preparations. Although some older adults may struggle to split tablets without tablet splitters, little evidence was found to justify tablet-splitting concerns other than the need to avoid splitting sustained-release preparations. With the exception of sustained-release medications, tablet splitting to facilitate lower medication doses and lower medication costs appears safe.

## Introduction

Using the lowest effective dose of all medications is key to minimising adverse drug effects in older adults and those with polypharmacy.^1,2^ Splitting tablets in half can help to achieve these lower doses, and often results in substantial cost savings for patients.^3–5^ However, manufacturers commonly discourage the splitting of their products, and this leads some healthcare providers to be reluctant to suggest it.^6^ To obtain more objective information on the safety of tablet splitting, a systematic review of the literature was conducted in which all arguments against tablet splitting, and all original research that validated or refuted those concerns, were gathered and synthesised.

## Method

### Review process

Dual reviewers were employed to evaluate titles and abstracts. A single reviewer assessed full texts for inclusion and extracted information. Two authors discussed and synthesised the data. As the primary interest was in gathering and synthesising both opinion and non-clinical trial research related to tablet splitting, the usual PRISMA processes for evaluating study quality (which focus on clinical trials) did not apply.

### Databases and search criteria

On 29 May 2019, Medline and EMBASE databases were searched for eligible studies spanning all available years of publication. With the assistance of a medical librarian searches were developed (see Supplementary Appendix S1) centred on the concepts of tablet (tablet*, pill, pills, and capsule) and splitting (split*, half, halv*, divid*, break*, cut, and cutting). The search was expanded under the heading of ’exp Tablets/ad [Administration & Dosage]’ and limited to the English language.

### Included studies

All studies discussing tablet splitting were obtained and read. This included original research, expert opinion, narrative review, and systematic review. Studies were excluded if tablet splitting was not a major focus of the article or if the article was not written in the English language.

## Results

**Figure 1.**
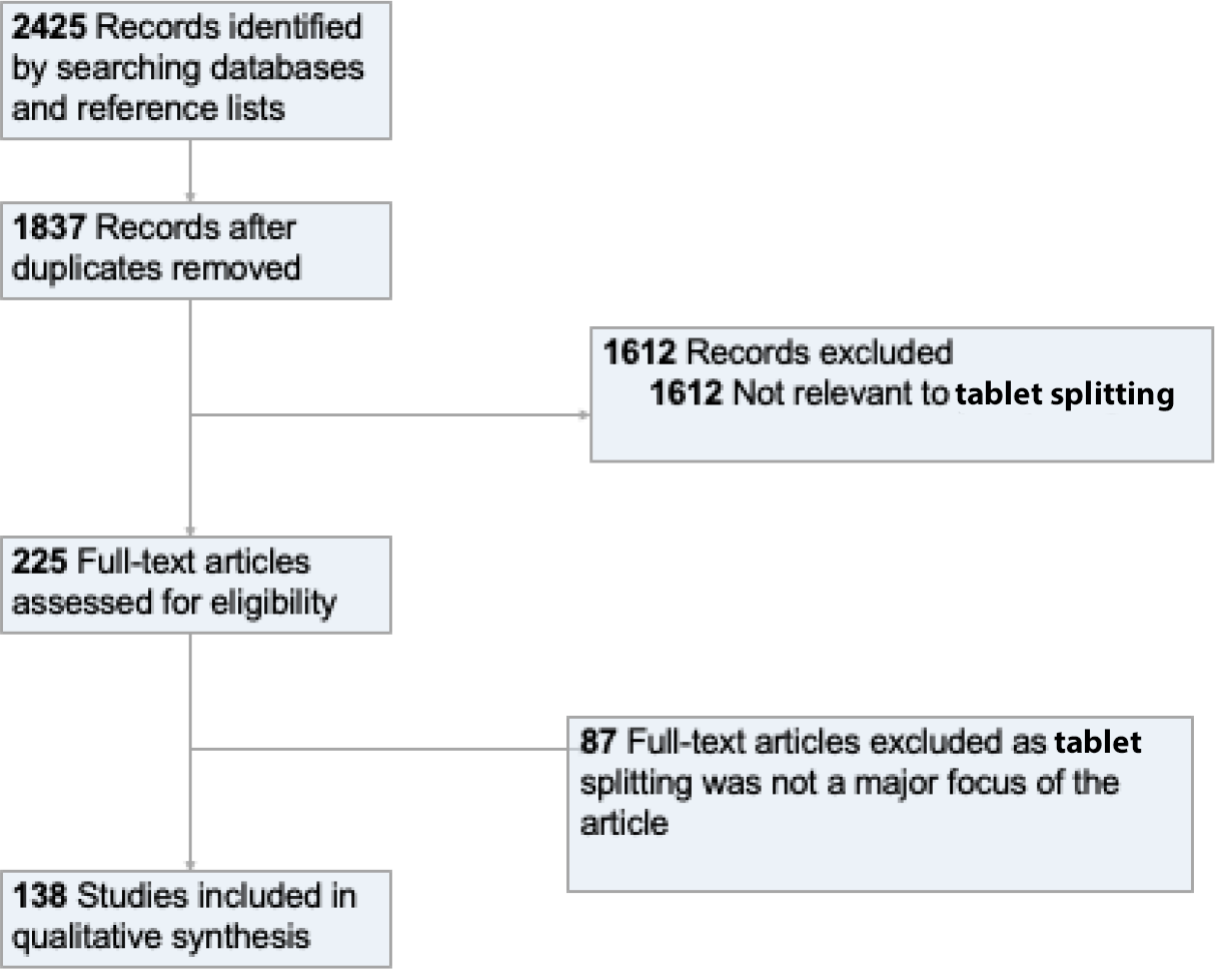
Study flow diagram

The database search yielded a total of 2425 articles, of which 588 were duplicates. After duplicates were removed, 1837 titles and abstracts were screened for inclusion. The full texts of 225 articles were examined, of which 138 met the inclusion criteria;^1–138^ these were included in the qualitative review (Figure 1). The characteristics of included publications are provided in Table 1. The list of included articles is available in the online supplement (see Supplementary Appendix S2), as is a table breaking each study down according to publication type and the concerns raised or addressed (see Supplementary Table S1).

**Table 1.** Characteristics of included publications (*n* = 138)

Publication type	*n* (%)
Original research	101 (73.2)
Opinion	32 (23.2)
Narrative review	4 (2.9)
Systematic review	1 (0.7)
Tablet-splitting advantages raised	
Cost savings	73 (52.9)
Dose flexibility/titration	66 (47.8)
Ease of swallowing	46 (33.3)
Tablet-splitting concerns raised	
Weight/dose variability	93 (67.4)
Difficulty breaking tablets	38 (27.5)
Loss of mass	29 (21.0)
Sustained-release tablets	27 (19.6)
Confusion/non-compliance	24 (17.4)
Chemical instability	19 (13.8)
Primary authors (first and last)^a^	
Pharmacist	128 (62.7)
Unknown	58 (28.4)
Specialist physician	9 (4.4)
Non-clinician	6 (2.9)
Generalist physician	3 (1.5)
Nurse	0 (0.0)
Location of first author	
North America	65 (47.1)
Western Europe	27 (19.6)
Asia	17 (12.3)
Eastern Europe	12 (8.7)
South America	7 (5.1)
Africa	5 (3.6)
Australia	5 (3.6)

^a^Denominator *n* = 204.

### Key concern 1: difficulty breaking tablets

Concern that patients may struggle to split tablets was raised in 38 articles,^1,3,5,7,8,11,14,16,27,34,39,43,46,50,51,54,72,91,92,95,98,99,103,105,107,108,110–113,120–122,124,125,128,134,135^ and pertained to both splitting by hand and the use of a tablet splitter. This concern focused on older adults potentially being limited by diminished manual dexterity or visual or cognitive impairments.^46,72,120^ Authors assumed tablets were harder to split when smaller, harder, and asymmetric in shape,^40^ and that splitting was easier using a tablet splitter than by hand. Score lines were expected to make splitting easier.^72^

### Evidence

#### Manual splitting

Of 120 older acute care patients admitted to a teaching hospital, 94 (78.3%) were unable to either break a scored tablet by hand unaided or to open a medication container manually.^139^ Manual splitting was similarly self-reported as being difficult by 29.7% of home-dwelling adults aged ≥70 years,^140^ and 36% of home-dwelling adults aged ≥75 years.^141^

#### Geometry and composition of the tablet matter

People who are diabetic aged >70 years were unable to split generic glyburide tablets 80% of the time, but only 30% failed to split a non-generic tablet with an easier to split design.^142^ Younger adults split tablets by hand more successfully than older adults (78.2% versus 38.1%), but age did not correlate with the accuracy of splitting tablets into equal halves when successfully split.^72^

#### Use of a tablet splitter

Numerous studies support tablet splitters making splitting easier.^3,4,93^ Of 233 responders to a survey (73% response rate) of Californian Air Force medical centre patients asked to split lovastatin tablets to reduce costs (mean age 65 years), only 6% felt the splitter was not easy to use.^143^ Similar results were reported for a convenience sample of 30 Dutch patients selected to have a wide variation in physical ability, and asked to split both a large and small round uncoated tablet. While 17% of all participants and 42% of those aged ≥65 years failed to split tablets by hand, all participants successfully split tablets using two types of splitter.^144^ In another convenience sample of 30 older adults (mean age 64.9 years), all successfully split a variety of tablets, although accurately splitting tablets into equal halves was better in those given instructions on splitter use.^65^ Overall, *evidence suggests splitting tablets by hand is challenging for some older adults, for whom tablet splitters, or assistance from pharmacists or family may be needed*.^39^

### Key concern 2: loss of mass

Concern that splitting could pulverise (turn to powder) a meaningful proportion of the tablet was raised in 29 articles.^1,2,9,13,28,29,31,34,43,46,50,57,72,74,77,81,82,92,101,110,118,121,122,124,125,128,133,134,137^ The resulting loss of mass could potentially lead to incorrect dosage, and contamination/health concerns for those unwittingly exposed to the residue.^72,121,128^ If tablets fragment to a large degree they may even need to be discarded, leading to increased healthcare costs.^64^

### Evidence

Although losses of mass up to 14% have been observed for tablets split into quarters,^127^ the average loss has been reported as 2.6% for round tablets, and as ‘insignificant‘ when tablets are oblong (an elongated oval shape).^145^ Multiple other studies describe loss of mass as acceptable or insignificant.^4,35,79^ Overall*, evidence suggests that loss of mass is negligible for the vast majority of medications*.

### Key concern 3: chemical instability

Concern that split tablets would chemically or physically degrade was raised in 19 articles.^5,21,26–28,36,39,42,50,52,54,70,79,82,83,98,103,118,128^ Concerns centred on increased friability of split tablets, and chemical reaction with air, water, or light once coatings were breached or packaging was removed. Splitting drugs with an enteric coating, used as a protective barrier against stomach acidity, can also increase the rate of degradation within the gut.^36,64,70,77,82^ Potential consequences could include patients experiencing more adverse effects, or receiving lower effective doses of the active substance.^35^

### Evidence

One study assessed chemical stability of 11 quartered cardiovascular medications stored in plastic containers without light exposure 30–45 days post-split. Three of these 11 medications demonstrated decreased levels of the active drug, including digoxin (for which mean drug concentration was 68% of expected), spironolactone (82%), and both generic and brand name amlodipine (91% and 93%, respectively). Only the drop in digoxin concentration was believed to be clinically important.^83^ Similarly, split tablets were considered chemically stable for levothyroxine (after 8 weeks at 25°C/60% relative humidity), aspirin (after 1 week of refrigeration), and gabapentin (after 9 weeks at room temperature).^36,54,146^ Another study found gabapentin, risperidone, and losartan to be chemically stable at 90 days (25°C ± 2°C/60% ± 5% relative humidity).^147^

More evidence on chemical stability of specific split medications is needed. However, *of the 16 studied medications identified, only digoxin degraded fast enough for chemical instability to be considered clinically important*. Where medications are known to degrade, or where there is uncertainty, splitting only one tablet at a time should mitigate this concern. No studies were found examining bioavailability after enteric coated tablets were split.

### Key concern 4: weight/dose variability

Concern that tablets would split unequally, and hence vary in dosage, was raised in 93 articles. This could lead to underdosing or overdosing, and was of particular concern for drugs with a narrow therapeutic index.^39,43^

### Evidence

Several studies evaluated split tablet weight variability.^22,41,79,83,110^ One study analysed 560 pharmacy-dispensed split tablets of 22 drugs, and found that only 32 (5.7%) of 560 tablet halves deviate >15% from the expected weight.^44^ In contrast, 41.3% of 1752 hydrochlorothiazide tablets manually split (that is, without a tablet splitter) by 94 healthy volunteers deviated from their expected weight by >10%.^79^ Other studies reported less clinically significant weight variation after splitting,^35,66^ including studies reporting the drug content of half-tablets of warfarin and salbutamol, split by tablet splitter, to fall within United States Pharmacopeia specification criteria.^89,93^ In a study of scored and unscored tablets of Risperdal, Paxil, and Zoloft split by tablet-splitter, all half-tablets produced uniform doses.^73^ Similarly, for 30 lorazepam half-tablets, drug content was within 75%–125% of expected for every half portion.^22^

Arguments against the clinical importance of weight variability, even where it does exist, were:

- average doses remain the same over time;
- differences in body weight are likely to have greater influence on drug levels than the observed minor differences in tablet weight;^148^ and
- a 10% variation in a single dose will not mean a 10% variation in steady state drug levels, especially for drugs with longer half-lives.^92,99^

In terms of efficacy, multiple studies found no significant changes in total or low-density lipoprotein cholesterol levels after splitting statins.^69,97,149^ Overall*, although minor weight variation is likely to occur to some extent, it is unlikely to be clinically important*.

### Key concern 5: sustained-release medications

Concern about splitting sustained-release medication was raised in 27 articles.^12,16,54,64,74,76,77,79,81,84,88,90,93,96,98,99,102–105,113–117,121,122^ Sustained release of medications with short half-lives is commonly achieved either by sustained-release external tablet coatings, or by embedding medication in a slowly degrading matrix. Splitting such preparations could compromise the release mechanism and result in overly rapid drug release and potential harm from overdose.^6,84,88,150,151^

### Evidence

While sustained-release preparations are not intended for splitting, it has been explored in some studies. Altered drug-release kinetics were reported post-split for sustained-release matrix tablets of diltiazem^116^ and aspirin,^84^ but not melatonin — although further cutting into quarters or crushing melatonin matrix tablets did alter release kinetics.^113^ Film-coated verapamil tablets also retained their release characteristics after splitting.^78^ Overall, while some sustained-release medications may be safe to split*, the impracticality of providers trying to remember which products can be split and which cannot, and the potential harm of overly rapid drug release, supports the generalisation against splitting all slow-release products*.^152^

### Key concern 6: confusion/non-compliance

Concern that directions to split tablets add complications that might confuse patients or lead to non-compliance was raised in 24 articles.^1,3,5,6,11,31,39,43,50,51,74,77,81,95,98,99,110–112,121,124,128,133,137^ In particular, there was concern that patients could split the wrong medication, that splitting could lead to miscommunication with patients or pharmacists (such as 1/2 tablet being misinterpreted to mean 1–2 tablets), or that the extra hurdle to use the medication could lead some to stop it altogether.

### Evidence

Using tablet counting and patient questionnaires, and tracking the patient refill history of 105 fosinopril users, there was no significant difference in compliance for those who split fosinopril tablets, and those who did not.^101^ This is consistent with reporting from a tablet-splitting programme, which reported no compliance problems.^143^ Another tablet-splitting programme involving 2019 patients reported that only 7% of patients found tablet splitting to influence their desire to take medication.^97^ No studies examining the frequency of errors being made by patients or pharmacists were found.

Overall, while evidence is needed to explore whether confusion or miscommunication can lead to medication errors, *available evidence suggests compliance does not suffer when tablets are split*. For patients where confusion may be more likely, this problem may be overcome by bubble-packing by pharmacists or weekly dosette box preparation by caregivers.

## Discussion

### Summary

Concerns related to tablet splitting include difficulty breaking tablets, loss of mass, weight/dose variability, chemical instability, disruption of sustained-release mechanisms, and confusion/non-compliance. Of these, evidence supports only the concern that some frail older adults may struggle to split tablets without a tablet splitter, and the caution that sustained-release tablets should not be split.

### Strengths and limitations

This study’s findings are strengthened by its having reviewed the literature systematically, with the use of dual reviewers in the review of titles and abstracts. It is limited by the qualitative nature of the synthesis, and the observational nature of much of the evidence. While including essentially all article types is a strength insofar as it enables a broad perspective of the topic, it is also a limitation, in that the tools and templates for reporting a study’s methods, findings, quality, and bias do not lend themselves to a collection of included studies that range from opinion to basic science.

### Comparison with existing literature

Taken together, the evidence gathered supports the experience of tablet-splitting programmes that describe splitting tablets as safe, effective, and readily accepted by patients and providers.^5,97^ The findings are at odds with narrative reviews that caution against tablet splitting.^98^

### Implications for practice

Provided that patients who would struggle to split tablets are assisted in doing so, and provided that sustained-release tablets are not targeted for splitting, tablet splitting appears to be an effective tool for utilising minimum effective doses and reducing medication costs.
